# Parental leave policy information during residency interviews

**DOI:** 10.1186/s12909-021-03067-y

**Published:** 2021-12-18

**Authors:** Molly B. Kraus, Emily G. Reynolds, Jillian A. Maloney, Skye A. Buckner-Petty, Julia A. Files, Sharonne N. Hayes, Cynthia M. Stonnington, Laura A. Vallow, Natalie H. Strand

**Affiliations:** 1 Department of Anesthesiology and Perioperative Medicine, Mayo Clinic, 5777 E Mayo Blvd, Phoenix, AZ 85054 USA; 2 Mayo Clinic Alix School of Medicine, Mayo Clinic, 13400 E Shea Blvd, Scottsdale, AZ 85259 USA; 3 Department of Quantitative Health Sciences, Mayo Clinic, 13400 E Shea Blvd, Scottsdale, AZ 85259 USA; 4 Department of Internal Medicine, Mayo Clinic, 13400 E Shea Blvd, Scottsdale, AZ 85259 USA; 5 Department of Cardiovascular Medicine, Mayo Clinic, 200 1st St SW, Rochester, MN 55905 USA; 6 Department of Psychiatry and Psychology, Mayo Clinic, 13400 E Shea Blvd, Scottsdale, AZ 85259 USA; 7 Department of Radiation Oncology, Mayo Clinic, 4500 San Pablo Road, Jacksonville, FL 32224 USA

**Keywords:** Medical education, Parental leave, Quality of life

## Abstract

**Background:**

During interviews, medical students may feel uncomfortable asking questions that might be important to them, such as parental leave. Parental leave policies may be difficult for applicants to access without asking the program director or other interviewers. The goal of this study is to evaluate whether parental leave information is presented to prospective residents and whether medical students want this information.

**Methods:**

Fifty-two program directors (PD’s) at 3 sites of a single institution received a survey in 2019 to identify whether parental leave information is presented at residency interviews. Medical students received a separate survey in 2020 to identify their preferences. Fisher exact tests, Pearson χ^2^ tests and Cochran-Armitage tests were used where appropriate to assess for differences in responses.

**Results:**

Of the 52 PD’s, 27 responded (52%) and 19 (70%) indicated that information on parental leave was not provided to candidates. The most common reason cited was the belief that the information was not relevant (n = 7; 37%). Of the 373 medical students, 179 responded (48%). Most respondents (92%) wanted parental leave information formally presented, and many anticipated they would feel extremely or somewhat uncomfortable (68%) asking about parental leave. The majority (61%) felt that these policies would impact ranking of programs “somewhat” or “very much.”

**Conclusions:**

Parental leave policies may not be readily available to interviewees despite strong interest and their impact on ranking of programs by prospective residents.

**Supplementary Information:**

The online version contains supplementary material available at 10.1186/s12909-021-03067-y.

## Introduction

Thousands of medical students interview for residency programs each year, and over 50% of medical students were women in 2019 [1]. The average age of US women having their first child is 26 years [2], which coincides with the beginning of residency for many trainees [3]. In a survey of 269 residency and fellowship programs, 40% of trainees indicated that they planned to have children during their graduate medical education (GME) [4].

A training program with a supportive culture can foster an ideal learning environment for residents [5]. Among residents planning to start or grow their family during training, the institution’s parental leave policy becomes an important part of the decision-making process when ranking residency programs [6]. The known benefits of paid parental leave include decreased infant mortality, improved physical and mental health for mother and infant, increased maternal participation in the labor force, and enhanced morale of parents [7–10]. However, parental leave policies vary widely between institutions [11]. In an analysis of the top 50 medical schools with affiliated GME programs, 42% had paid leave policies for residents and fellows (average duration, 5.1 weeks), 42% did not provide any paid leave, and 22% offered state-mandated, partially paid leave [12].

During residency interviews, presentations of parental leave policies may also vary, and prospective residents may not inquire directly about these policies despite their interest, because of perceived potential negative consequences. These fears of stigma are not unfounded. In various surveys of PD’s, 61–83% have reported a belief that becoming a parent during residency negatively affects the performance of female physicians [7, 13].

Family and lifestyle factors are among the most influential considerations for medical students as they plan their lives and careers [14], so it is important for parental leave policies to be presented uniformly and consistently during the interview process. We sought to investigate whether parental leave policies are presented during residency interviews and to evaluate whether medical students are interested in that information.

## Methods

An electronic survey for PD’s was developed to determine whether parental leave policies were presented during residency interviews (Supplemental Digital Appendix 1). The voluntary electronic survey was emailed in November 2019 to all PD’s within our institution, with training sites in 3 states. PD’s were asked whether they addressed parental leave policies at interviews and why or why not. Multiple responses could be selected, and free text responses were later grouped according to similarity. Responses were compared by respondents’ sex and age (< 50 years or ≥ 50 years).

An electronic survey for medical students was created to determine whether parental leave policies were presented at residency interviews (Supplemental Digital Appendix 2). The voluntary electronic survey was emailed in June 2020 to medical students at our institution, which has 1 medical school and 3 sites. Results were analyzed according to sex, age, marital status, current parental status, and reproductive plans during residency.

Responses were summarized as frequencies and percentages. Fisher exact tests were used to assess for group differences in the responses from PD’s. Differences in responses from medical students were assessed with Pearson χ^2^ tests for nominal variables and Cochran-Armitage tests for Likert scale variables. In addition, Cramer’s V and Cliff’s Delta effect sizes were computed to show association strength for nominal variables and Likert scale variables respectively. Association strength based on Cramer’s V is considered negligible if less than 0.1; small if between 0.1 and 0.3; medium if between 0.3 and 0.5; and large if 0.5 or greater [15]. Association strength based on Cliff’s Delta is considered negligible if the magnitude is less than 0.147; small if between 0.147 and 0.33; medium if between 0.33 and 0.474; and large if 0.474 or greater [16]. Two-sided *P* values less than 0.05 were considered statistically significant. All statistical analyses were conducted with R version 3.6 software (R Foundation) [17]. Institutional review was sought, and the project was deemed exempt.

## Results

Of the 52 PD’s who were surveyed, 27 responded (52%). A majority (n = 19; 70%) indicated that parental leave was not addressed with candidates for reasons that included the following: information was not relevant (n = 7; 37%); candidates were not interested (n = 2; 11%); policy was addressed elsewhere (n = 4; 21%); residents should not have children during training (n = 1; 5%); policy was provided only upon request (n = 3; 16%); and insufficient time (n = 3; 16%) (Table 1). Presentation methods varied amongst the 8 PD’s who presented policy information and included slide presentation (n=4HR presentation (n=1) discussion if asked (n=4), discussed with all candidates (n=3). There were no statistically significant differences in PD’s responses when evaluated by sex or age.

**Table 1:** Program Director (PD) Survey Results (N = 27^a^)

Item	n (%)
**What is your gender identity? (n = 23)**	
Female	8 (35)
Male	15 (65)
**What is your age group? (n = 24)**	
30–39 y	2 (8)
40–49 y	15 (63)
50–59 y	5 (21)
60–69 y	2 (8)
**Are parental leave policies addressed with candidates during the residency interview process?**	
No	19 (70)
Yes	8 (30)
**How are parental leave policies addressed during the residency interview process? Select all that apply (n = 8)**	
Verbally discussed with all candidates	3 (38)
Discussed only if asked by a candidate	4 (50)
Addressed with HR	1 (13)
Presented formally in slide presentation	4 (50)
Other	1 (13)
**Why are residency parental leave policies not addressed during the interview process? Select all that apply (n = 19)**	
I believe it is being presented elsewhere	4 (21)
I do not believe it is relevant to the interview process	7 (37)
I do not believe candidates are interested in the program’s parental leave policy	2 (11)
I do not believe residents should be having children during training	1 (5)
Other (free text response)	12 (63)
Not addressed in interview process	5 (26)
Address if asked	3 (16)
No time	3 (16)
Blank	1 (5)

^a^ Smaller sample sizes (eg, because of no response) are indicated for specific item

The medical student survey was sent to 373 students throughout all 4 years, and 179 responded (48%). Demographics are summarized in Supplemental Digital Appendix 3. Most respondents did not have children (n = 155, 87%). More than half (n = 111, 62%) anticipated that they or their partner would have a child during residency. The majority (n = 108, 92%) would appreciate having information related to parental leave formally presented at residency interviews. Many respondents reported that they would anticipate feeling at least somewhat uncomfortable (n = 119, 68%) asking about parental leave at an interview. Of those, 59% (n = 105) worried it would lessen their chance of acceptance. There was no consensus regarding whether or not they expect parental leave policies to be formally presented. When asked whether a parental leave policy would impact their ranking of programs, 78% (n = 135) responded that it would “somewhat” or “very much” affect their decision on ranking otherwise equivalent programs. When asked how they would like to receive information on parental leave policy, 76% (n = 133) desired formal presentation; 57% (n = 101) desired handouts during the interview; and 39% (n = 69) desired unofficial conversations. In regard to whether or not fourth-year students who had already interviewed (n = 24) received this information formally, 14 (58%) responded, “sometimes”; 8 (33%), “never”; and 2 (8%), “always.” When asked whether leave policies were informally discussed, 17 (71%) responded, “sometimes”; 5 (21%), “never”; and 2 (8%), “always.” When comparing respondents according to sex and response to the question “Do you anticipate you or your partner will have a child during residency,” significant differences were noted for several responses (Tables 2 and 3). Three respondents did not provide their sex in the survey and were excluded from Table 2 (n = 176). While the large majority of men and women indicated that they would appreciate formal presentation of parental leave information, women responded positively at a significantly higher rate (96% vs 84%; Cramer’s V = 0.25; *P* = 0.004). Women were significantly more uncomfortable asking the PD about information related to parental leave (80% vs 46%; Cliff’s Delta = -0.39; *P* < 0.001). A higher percentage of women also responded that these policies would impact their ranking of residency programs. Respondents who anticipated that they or their partner would have a child during residency reported that a difference in parental leave policy may somewhat (n = 42; 39%) or very much (n = 52; 48%) impact their ranking of 2 equivalent programs (Table 3). We found no significant differences in responses according to age, marital status, or current parental status.

**Table 2:** Medical Student Responses to Questions About Parental Leave According to Sex of Respondent

**Item**	**Female n (%) (n = 113)**		**Male n (%) (n = 63)**	**Total n (%) (n = 176**^c^**)**	***P* value**	**Effect** **Size**
**Do you expect parental leave policies will be formally presented at residency interviews?**					.20^a^	
Definitely not		2 (1.8)	2 (3.2)	4 (2.3)		
Probably not		22 (19.5)	18 (28.6)	40 (22.7)		
Might or might not		34 (30.1)	14 (22.2)	48 (27.3)		
Probably yes		28 (24.8)	19 (30.2)	47 (26.7)		
Definitely yes		27 (23.9)	10 (15.9)	37 (21.0)		
**Would you appreciate if information related to parental leave was formally presented at residency interviews?**					.004 ^b^	.25
Don’t care		2 (1.8)	9 (14.3)	11 (6.2)		
No		3 (2.7)	1 (1.6)	4 (2.3)		
Yes		108 (95.6)	53 (84.1)	161 (91.5)		
**During interviews, do you anticipate you would be comfortable asking the PD about the parental leave policy?**					< .001^a^	-.39
Extremely uncomfortable		30 (26.5)	5 (7.9)	35 (19.9)		
Somewhat uncomfortable		60 (53.1)	24 (38.1)	84 (47.7)		
Neither comfortable nor uncomfortable		7 (6.2)	15 (23.8)	22 (12.5)		
Somewhat comfortable		14 (12.4)	14 (22.2)	28 (15.9)		
Extremely comfortable		2 (1.8)	5 (7.9)	7 (4.0)		
**Why do you anticipate you would be uncomfortable? Select all that apply**						
Awkward topic		14 (12.4)	5 (7.9)	19 (10.8)		
Power imbalance (candidate asking PD or other physician)		0 (0.0)	0 (0.0)	0 (0.0)		
I am worried it will lessen my chance of acceptance		78 (69.0)	27 (42.9)	105 (59.7)		
Don’t want to talk about leave at an interview		52 (46.0)	19 (30.2)	71 (40.3)		
Other		2 (1.8)	2 (3.2)	4 (2.3)		
**When evaluating residency programs, do you anticipate parental leave policy will impact your ranking of programs? (N = 172)**					.003 ^b^	.29
Not at all		16 (14.7)	25 (39.7)	41 (23.8)		
Somewhat		52 (47.7)	23 (36.5)	75 (43.6)		
Very much		22 (20.2)	7 (11.1)	29 (16.9)		
Unsure		19 (17.4)	8 (12.7)	27 (15.7)		
**Given 2 equivalent programs, would a difference in parental leave policy impact your decision on ranking? (N = 173)**					.002 ^b^	.29
Not at all		5 (4.5)	12 (19.0)	17 (9.8)		
Somewhat		40 (36.4)	29 (46.0)	69 (39.9)		
Very much		51 (46.4)	15 (23.8)	66 (38.2)		
Unsure		14 (12.7)	7 (11.1)	21 (12.1)		
**How would you like to receive information about parental leave during the residency application? Select all that apply**						
During the formal presentation about the program at the time of the interview		90 (79.6)	43 (68.3)	133 (75.6)		
Handouts distributed to me at the time of the interview		69 (61.1)	32 (50.8)	101 (57.4)		
Training program’s website (available to applicants)		0 (0.0)	0 (0.0)	0 (0.0)		
Personal unofficial conversations with current residents		46 (40.7)	23 (36.5)	69 (39.2)		
**Were parental leave policies formally discussed or presented during residency interviews? (N = 32)**					.42 ^b^	
Never		4 (26.7)	4 (44.4)	8 (33.3)		
Sometimes		9 (60.0)	5 (55.6)	14 (58.3)		
Always		2 (13.3)	0 (0.0)	2 (8.3)		
**Were parental leave policies informally discussed during residency interviews? (N = 32)**					.31 ^b^	
Never		2 (13.3)	3 (33.3)	5 (20.8)		
Sometimes		11 (73.3)	6 (66.7)	17 (70.8)		
Always		2 (13.3)	0 (0.0)	2 (8.3)		

^a^ Cochran-Armitage trend test for ordinal variables. Associated effect size is Cliff’s Delta

^b^ Pearson χ^2^ test. Associated effect size is Cramer’s V

^c^ Smaller sample sizes (because of no response or smaller survey group) are indicated for specific items

**Table 3:** Medical Student Responses According to Answers to the Question “Do You Anticipate You or Your Partner Will Have a Child During Residency?”

Item	No n (%) (n = 67)	Yes n (%) (n = 111)	Total n (%) (n = 178^c^)	*P* value	Effect Size
**Do you expect parental leave policies will be formally presented at residency interviews?**				.71^a^	
Definitely not	1 (1.5)	3 (2.7)	4 (2.2)		
Probably not	18 (26.9)	24 (21.6)	42 (23.6)		
Might or might not	17 (25.4)	31 (27.9)	48 (27.0)		
Probably yes	18 (26.9)	29 (26.1)	47 (26.4)		
Definitely yes	13 (19.4)	24 (21.6)	37 (20.8)		
**Would you appreciate if information related to parental leave was formally presented at residency interviews?**				.74 ^b^	
Don’t care	5 (7.5)	6 (5.4)	11 (6.2)		
No	2 (3.0)	2 (1.8)	4 (2.2)		
Yes	60 (89.6)	103 (92.8)	163 (91.6)		
**During interviews, do you anticipate you would be comfortable asking the PD about the parental leave policy?**				.44^a^	
Extremely uncomfortable	17 (25.4)	19 (17.1)	36 (20.2)		
Somewhat uncomfortable	28 (41.8)	56 (50.5)	84 (47.2)		
Neither comfortable nor uncomfortable	11 (16.4)	11 (9.9)	22 (12.4)		
Somewhat comfortable	8 (11.9)	21 (18.9)	29 (16.3)		
Extremely comfortable	3 (4.5)	4 (3.6)	7 (3.9)		
**Why do you anticipate you would be uncomfortable? Select all that apply**					
Awkward topic	5 (7.5)	14 (12.6)	19 (10.7)		
Power imbalance (candidate asking PD or other physician)	0 (0.0)	0 (0.0)	0 (0.0)		
I am worried it will lessen my chance of acceptance	37 (55.2)	69 (62.2)	106 (59.6)		
Don’t want to talk about leave at an interview	27 (40.3)	44 (39.6)	71 (39.9)		
Other	2 (3.0)	2 (1.8)	4 (2.2)		
**When evaluating residency programs, do you anticipate parental leave policy will impact your ranking of programs? (N = 174)**				< .001 ^b^	.33
Not at all	22 (33.3)	19 (17.6)	41 (23.6)		
Somewhat	27 (40.9)	49 (45.4)	76 (43.7)		
Very much	2 (3.0)	27 (25.0)	29 (16.7)		
Unsure	15 (22.7)	13 (12.0)	28 (16.1)		
**Given 2 equivalent programs, would a difference in parental leave policy impact your decision on ranking the programs? (N = 175)**				.001 ^b^	.30
Not at all	9 (13.6)	8 (7.3)	17 (9.7)		
Somewhat	28 (42.4)	42 (38.5)	70 (40.0)		
Very much	15 (22.7)	52 (47.7)	67 (38.3)		
Unsure	14 (21.2)	7 (6.4)	21 (12.0)		
**How would you like to receive information about parental leave during the residency application?**					
During the formal presentation about the program at the time of the interview	48 (71.6)	87 (78.4)	135 (75.8)		
Handouts distributed to me at the time of the interview	35 (52.2)	68 (61.3)	103 (57.9)		
Training program’s website (available to applicants)	0 (0.0)	0 (0.0)	0 (0.0)		
Personal unofficial conversations with current residents	25 (37.3)	44 (39.6)	69 (38.8)		
**Were parental leave policies formally discussed or presented during residency interviews? (N = 32)**				.16 ^b^	
Never	2 (20.0)	6 (42.9)	8 (33.3)		
Sometimes	8 (80.0)	6 (42.9)	14 (58.3)		
Always	0 (0.0)	2 (14.3)	2 (8.3)		
**Were parental leave policies informally discussed during residency interviews? (N = 32)**				.54 ^b^	
Never	1 (10.0)	4 (28.6)	5 (20.8)		
Sometimes	8 (80.0)	9 (64.3)	17 (70.8)		
Always	1 (10.0)	1 (7.1)	2 (8.3)		

^a^ Cochran-Armitage trend test for ordinal variables. Associated effect size is Cliff’s Delta

^b^ Pearson χ^2^ test. Associated effect size is Cramer’s V

^c^ Smaller sample sizes (because of no response or smaller survey group) are indicated for specific items

## Discussion

We found that nearly all residency applicants (92%) would prefer parental leave policies to be formally addressed during interview. Over 60% of respondents anticipated having a child during residency, thus making parental leave policies highly relevant. The discomfort expressed by medical students, highest amongst women, with asking about these policies is in direct contrast with the fact that 70% of PD’s responded that parental leave is not formally presented. 69% of women and 43% of men reported that they were worried asking about parental leave during the interview would lessen their chance of acceptance into the program. This discordance between applicants’ needs and current training program practice represents a gap and an opportunity. Some PD’s responded that if asked, they would address parental leave, unveiling the lack of awareness many may have regarding the comfort of medical students addressing these subjects.

In the 2017–2018 medical school application cycle, the average age of male and female applicants was 24 years, which positions most graduating medical students in their childbearing years during residency [3]. A survey of 705 medical students interested in family medicine residencies demonstrated that work-life balance was among the highest rated factors influencing residency selection [14]. Another survey of 644 residents and fellows found that approximately 40% planned to have children during their GME [4]. The same survey found that women were more likely than men to consider pregnancy and childbirth in their career decisions about GME programs, completion dates, and other degrees. In a survey of pediatric residents with children, over half of women and a third of men rated parental leave policies as very important in residency selection [18].

The Accreditation Council for Graduate Medical Education (ACGME) does not have a universal parental leave policy that must be followed by all GME programs [19]. The ACGME requires each GME program to have a leave policy according to their specialty board, but there is no clear consensus on how these policies should be presented or made available to applicants. This ambiguity presents a challenge for medical students who are considering childbearing during their GME. A study of the policies of 24 American Board of Medical Specialties organizations found that not all specialty boards outline family leave policies and that many policies have vague language [20]. Given the complexity of leave policies, clarification may be important to a large proportion of medical students, which is confirmed in our study.

Our findings suggest that parental leave policies should be presented formally to all candidates during the interview process to better address applicant needs, relieve the burden of having to ask and to mitigate the risk of negative bias toward applicants who want that information. More than half the surveyed medical students stated that parental leave policies would impact how they ranked programs, and would impact their decision if they were deciding between 2 equivalent programs. By formally presenting the information during interviews, PD’s can facilitate a better match and alignment of priorities for both the applicant and the program. As the number of women applying to medical schools increases, programs interested in attracting the best applicants would be wise to acknowledge the importance of parental leave policies to students who are ranking their programs.

Our study is limited by the small number of PD’s who responded and by the survey being conducted at a single institution. Larger multisite surveys of both PD’s and medical students may further elucidate the importance of parental leave policies on applicants’ selection of a residency program.

## Conclusion

A gap exists between 1) medical student interest and comfort level with obtaining information about parental leave policies and 2) residency PD’s perception of the importance and relevance of this information. Parental leave policies are important to medical students interviewing for residency, but they may not be regularly shared or easily accessible. Parental leave policies and practices should be formally presented to all residency candidates before or during the interview process.

## Supplementary Information

**Additional file 1: Digital Appendix 1.** Program Director Survey on Parental Leave Policies at Residency Interviews

**Additional file 2: Digital Appendix 2.** Medical Student Survey on Parental Leave Policies at Residency Interviews

**Additional file 3: Digital Appendix 3. **Demographics of Medical Student Respondents

## Data Availability

The datasets used and/or analyzed during the current study are available from the corresponding author on reasonable request.

## Abbreviations

ACGME: Accreditation Council for Graduate Medical Education
GME: Graduate medical education
PD: Program director
