# Correction to: Parental leave policy information during residency interviews

**DOI:** 10.1186/s12909-022-03114-2

**Published:** 2022-01-25

**Authors:** Molly B. Kraus, Emily G. Reynolds, Jillian A. Maloney, Skye A. Buckner-Petty, Julia A. Files, Sharonne N. Hayes, Cynthia M. Stonnington, Laura A. Vallow, Natalie H. Strand

**Affiliations:** 1 Department of Anesthesiology and Perioperative Medicine, Mayo Clinic, 5777 E Mayo Blvd, Phoenix, AZ 85054 USA; 2 Mayo Clinic Alix School of Medicine, Mayo Clinic, 13400 E Shea Blvd, Scottsdale, AZ 85259 USA; 3 Department of Quantitative Health Sciences, Mayo Clinic, 13400 E Shea Blvd, Scottsdale, AZ 85259 USA; 4 Department of Internal Medicine, Mayo Clinic, 13400 E Shea Blvd, Scottsdale, AZ 85259 USA; 5 Department of Cardiovascular Medicine, Mayo Clinic, 200 1st St SW, Rochester, MN 55905 USA; 6 Department of Psychiatry and Psychology, Mayo Clinic, 13400 E Shea Blvd, Scottsdale, AZ 85259 USA; 7 Department of Radiation Oncology, Mayo Clinic, 4500 San Pablo Road, Jacksonville, FL 32224 USA

**Correction to: BMC Medical Education (2021) 21:623**

**https://doi.org/10.1186/s12909-021-03067-y**

Following publication of the original article [1], we have been informed that the authors Jillian A. Maloney, Skye A. Buckner-Petty and Natalie H. Strand were incorrectly affiliated. Also, the affiliation address for the author Sharonne N. Hayes was incorrect.

The authors affiliation has been updated above and the original article [1] has been corrected.

The publishers apologise for this error.
