# Supplementary material for: Parental leave policy information during residency interviews
Source: BMC Med Educ. 2021 Dec 18;21:623. doi: 10.1186/s12909-021-03067-y (PMC8684616; doi:10.1186/s12909-021-03067-y)
Supplement: Supplementary file 1 — Additional file 1: Digital Appendix 1. Program Director Survey on Parental Leave Policies at Residency Interviews [file 12909_2021_3067_MOESM1_ESM.docx]

Supplemental Digital Appendix 1. Program Director Survey on Parental Leave Policies at Residency Interviews

Start of Block: Mayo Clinic

Q3 Are you a residency program director?

- Yes (1)
- No (2)

Display This Question:

If Are you a residency program director? = Yes

Q1 Are parental leave policies addressed with candidates during the residency interview process?

- Yes (1)
- No (2)

Display This Question:

If Are parental leave policies addressed with candidates during the residency interview process? = Yes

Q2 How are parental leave policies addressed during residency interview process? Select all that apply

- Presented formally in powerpoint (1)
- Given to all candidates in a hand out (2)
- Verbally discussed with all candidates (3)
- Discussed only if asked by a candidate (4)
- Addressed with HR (5)
- Other (6) ________________________________________________

Display This Question:

If Are parental leave policies addressed with candidates during the residency interview process? = No

Q4 Why are residency parental leave policies not addressed during the interview process? Select all that apply

- I believe it is being presented elsewhere (1)
- I do not believe it is relevant to the interview process (2)
- I do not believe candidates are interested in the program's parental leave policy (3)
- I do not believe residents should be having children during training (4)
- Other (5) ________________________________________________

| Page Break |  |
| --- | --- |

Display This Question:

If Are you a residency program director? = Yes

Q5 What is your gender identity?

- Male (1)
- Female (2)
- Other (3)

Display This Question:

If Are you a residency program director? = Yes

Q6 What is your age group?

- under 30 (1)
- 30-39 (2)
- 40-49 (3)
- 50-59 (4)
- 60-69 (5)
- 70 and older (6)

End of Block: Mayo Clinic

© Mayo Foundation for Medical Education and Research.
