# Supplementary material for: Parental leave policy information during residency interviews
Source: BMC Med Educ. 2021 Dec 18;21:623. doi: 10.1186/s12909-021-03067-y (PMC8684616; doi:10.1186/s12909-021-03067-y)
Supplement: Supplementary file 2 — Additional file 2: Digital Appendix 2. Medical Student Survey on Parental Leave Policies at Residency Interviews [file 12909_2021_3067_MOESM2_ESM.docx]

Supplemental Digital Appendix 2. Medical Student Survey on Parental Leave Policies at Residency Interviews

Start of Block: Default Question Block

Q1 Year in medical school

- M1 (1)
- M2 (2)
- M3 (3)
- M4 (4)
- Other (5) ________________________________________________

Q2 Age at anticipated medical school graduation

________________________________________________________________

Q3 Which sex were you assigned at birth?

- Male (1)
- Female (2)
- Prefer not to disclose (3)

Q4 To which residency program(s) will you apply?

- Unsure/ Undecided (1)
- Anesthesiology (2)
- Dermatology (3)
- Emergency Medicine (4)
- Family Medicine (5)
- General Surgery (6)
- Internal Medicine (7)
- Neurology (8)
- Neurosurgery (9)
- OBGYN (10)
- Otolaryngology (11)
- Ophthalmology (12)
- Orthopedic Surgery (13)
- Pathology (14)
- Pediatrics (15)
- Plastic surgery (16)
- PM&R (17)
- Preventative Medicine (18)
- Psychiatry (19)
- Radiology (20)
- Radiation Oncology (21)
- Urology (22)
- Other (23) ________________________________________________

Q15 What is your marital status?

- Married (1)
- Single (2)
- Engaged (3)
- Prefer not to disclose (4)
- Committed long term relationship (5)

Q5 Do you have children?

- Yes (1)
- No (2)

Q6 Do you anticipate you or your partner will have a child during residency?

- Yes (1)
- No (2)

Q8 Do you expect parental leave policies will be formally presented at residency interviews?

- Definitely yes (1)
- Probably yes (2)
- Might or might not (3)
- Probably not (4)
- Definitely not (5)

Q9 Would you appreciate if information related to parental leave was presented at residency interviews?

- Yes (1)
- No (2)
- Don't care (3)

Q7 During interviews, do you anticipate you would be comfortable asking the program director about the parental leave policy?

- Extremely comfortable (1)
- Somewhat comfortable (2)
- Neither comfortable nor uncomfortable (3)
- Somewhat uncomfortable (4)
- Extremely uncomfortable (5)

Display This Question:

If During interviews, do you anticipate you would be comfortable asking the program director about t... = Somewhat uncomfortable

Or During interviews, do you anticipate you would be comfortable asking the program director about t... = Extremely uncomfortable

Q10 Why do you anticipate you would be uncomfortable? Select all that apply

- Awkward topic (1)
- Don't want to talk about leave at an interview (2)
- Power imbalance (3)
- I am worried it will lessen my chance of acceptance (4)
- Other (5) ________________________________________________

Q11 When evaluating residency programs, do you anticipate parental leave policy will impact your ranking of programs?

- Very much (1)
- Somewhat (2)
- Not at all (3)
- Unsure (4)

Q12 Given 2 equivalent programs, would a difference in parental leave policy impact your decision on ranking the programs?

- Very much (1)
- Somewhat (2)
- Not at all (3)
- Unsure (4)

Display This Question:

If Would you appreciate if information related to parental leave was presented at residency interviews? = Yes

Q13 How would you like to receive information about parental leave during the residency application  process? (Select all that apply)

- During the formal presentation about the program at the time of the interview (1)
- Handouts distributed to me at the time of the interview (2)
- Personal unofficial conversations with current residents (3)
- Training program's website (available to applicants) (4)

End of Block: Default Question Block

© Mayo Foundation for Medical Education and Research.
