# Supplementary material for: Parental leave policy information during residency interviews
Source: BMC Med Educ. 2021 Dec 18;21:623. doi: 10.1186/s12909-021-03067-y (PMC8684616; doi:10.1186/s12909-021-03067-y)
Supplement: Supplementary file 3 — Additional file 3: Digital Appendix 3. Demographics of Medical Student Respondents [file 12909_2021_3067_MOESM3_ESM.docx]

**Supplemental Digital Appendix 3.** Demographics of Medical Student Respondents

**(N=179^*^)**

| **Item** | **No (%)**^†^ |  | **No (%)**^†^ |  | **No (%)**^†^ |
| --- | --- | --- | --- | --- | --- |
| **Year in medical school (during 2019-2020 academic year)** | | | |  |  |
| 1 | 44 (24.6) |  |  |  |  |
| 2 | 42 (23.5) |  |  |  |  |
| 3 | 57 (31.8) |  |  |  |  |
| 4 | 32 (17.9) |  |  |  |  |
| Other | 4 (2.2) |  |  |  |  |
| **Age at anticipated medical school graduation?** | | | |  |  |
| Median (Q1, Q3) | 27 (26, 29) |  |  |  |  |
| Range | 22-41 |  |  |  |  |
| **Which sex were you assigned at birth? (n=176)** | | | |  |  |
| Female | 113 (64.2) | Male | 63 (35.8) |  |  |
| **To which residency program(s) will you apply?** | | | |  |  |
| Anesthesiology | 14 (7.8) | OB-GYN | 11 (6.1) | Preventative medicine | 1 (0.6) |
| Dermatology | 12 (6.7) | Ophthalmology | 9 (5.0) | Psychiatry | 13 (7.3) |
| Emergency medicine | 22 (12.3) | Orthopedic surgery | 16 (8.9) | Radiation oncology | 5 (2.8) |
| Family medicine | 10 (5.6) | Otolaryngology | 6 (3.4) | Radiology | 5 (2.8) |
| General surgery | 17 (9.5) | Pathology | 3 (1.7) | Urology | 5 (2.8) |
| Internal medicine | 33 (18.4) | Pediatrics | 17 (9.5) | Unsure or undecided | 44 (24.6) |
| Neurology | 7 (3.9) | Plastic surgery | 7 (3.9) | Other | 3 (1.7) |
| Neurosurgery | 4 (2.2) | PM&R | 1 (0.6) |  |  |
| **What is your marital status? (n=177)** | | | |  |  |
| Engaged | 9 (5.1) | Married | 48 (27.1) |  |  |
| Long-term committed relationship | 43 (24.3) | Single | 77 (43.5) |  |  |
| **Do you have children? (n=178)** | | | |  |  |
| No | 155 (87.1) | Yes | 23 (12.9) |  |  |
|  |  |  |  |  |  |
| **Do you anticipate you or your partner will have a child during residency? (n=178)** | | | |  |  |
| No | 67 (37.6) | Yes | 111 (62.4) |  |  |

Abbreviations: OB-GYN = obstetrics-gynecology; PM&R =

physical medicine and rehabilitation; Q = quartile.

^*^ Smaller sample sizes (eg, because of no response) are indicated

for specific items.

^†^ Values are presented as number (percentage) unless indicated otherwise
